# 

**Published:** 1896-03-28

**Authors:** 


### EPILEPTICS IN AMERICA.

The Pennsylvania Epileptic Hospital and the Colony Farm for Epileptics have been amalgamated and will henceforth work in unison, patients being transferred from one to the other as found desirable.

**IMPORTANT NOTICE.**

On account of the Easter Holidays, we beg to announce that we shall go to press with our issue of the 4th APRIL on TUESDAY NIGHT, the 31st INST. Advertisements intended for insertion in this issue should therefore reach us BY NOON ON TUESDAY, the 31st inst.

**Notices of Births, Deaths, and Marriages** are inserted in this column at a charge of 2s. 6d. for an announcement not exceeding four lines.

**Notice to Advertisers and Subscribers.**
All Advertisements, Orders for Copies of The Hospital and Business Communications should be addressed to **THE MANAGER, (Not to The Editor)**, The Hospital, 428, Strand, London, W.C.
The Cost of Subscription, post free, is as follows:—Per week, 2½d.; per quarter, 2s. 8d.: per half-year, 5s. 3d.; per annum, 10s. 6d. Foreign:—Per Annum 15s. 2d.; payable, in all cases, in advance.

### NOTICE TO CORRESPONDENTS.

All MS., Letters, Books for Review, and other matters intended for the Editor should be addressed The Editor, The Lodge, Porchester Square, London, W.

The Editor cannot undertake to return rejected MS., even when accompanied by stamped directed envelope.

**"Burdett's Hospitals and Charities."**
Sixth year of publication. 950 pp. Scarlet cloth, gilt lettered. Price 5s. A most complete guide to British, Colonial, Indian, and American Hospitals, Dispensaries, Nursing and Convalescent Institutions, and Asylums. A few complete sets of the preceding five issues of the "Annual" may still be obtained on application to the Publishers, The Scientific Press (Limited), 428, Strand, London, W.C.

**NOTICE.—Vol. XIX. of "The Hospital" (October, 1895, to March, 1896), in handsome cloth binding, will be on sale at the Office of this Journal early in April. Price 7s. 6d. Cases for binding the Numbers contained in this Volume, 1s. 6d. Index to Vol. XIX., 2½d., post free.**

**The Monthly Part for April will be ready next week, price 6d., by post, 8d.**

### The Student of Medicine.

### APPOINTMENTS.

W. S. Batten, M.R.C.S.Eng., L.S.A., appointed Medical Officer for the Second District of the Calne Union. E. Wharmby Battle, M.R.C.S.Eng., L.R.C.P.Lond., appointed Junior House Surgeon to the Warrington Infirmary. R. W. Beesley, M.B., C.M.M.Edin., appointed Junior House Surgeon to the Bolton Infirmary and Dispensary. G. F. Blacker, M.D., B.S., M.R.C.P., F.R.C.S., appointed Obstetric Physician to Out-patients, Great Northern Central Hospital. R. O. Bowman, M.D.Lond., L.R.C.P., M.R.C.S., appointed Medical Officer for the Ulverston District of the Ulverston Union. W. Bulloch, M.D., appointed Director of the Curative Serum Department of the British Institute of Preventive Medicine. A. W. Collins, M.B.Vict., L.R.C.P.Lond., appointed Medical Officer for the Workhouse of the Ulverston Union. J. R. Crease, jun., L.R.C.P., L.R.C.S. Edin., appointed House Surgeon to the Sunderland and North Durham Eye Infirmary. Adèle Isabella De Steiger, M.B.Lond., appointed Third Assistant Medical Officer to the Essex County Lunatic Asylum, Brentwood. J. D. Duncan, M.B., C.M.Edin., appointed Junior House Surgeon to the Infirmary, Carlisle. L. M. Dunlop, L.R.C.P., L.R.C.S.Edin., appointed Medical Officer to the Casual Wards of the Bow Workhouse. J. Edgar, M.A., B.Sc., M.B., C.M., appointed Professor of Midwifery at Anderson's College Medical School, Glasgow. C. W. Graham, F.F.P.S.Glasg., L.R.C.P.I., appointed Honorary Surgeon to the Carlisle Dispensary. S. A. Jolly, L.R.C.P., L.R.C.S.Edin., appointed Medical Officer for the Acton District of the Brentford Union. H. Dove King, M.D., M.A., B.Sc.Edin., appointed Certifying Factory Surgeon for Sudbury. J. W. Maskell, M.R.C.S., L.R.C.P.Lond., appointed Junior House Surgeon to the Bootle Borough Hospital. A. F. Messiter, M.R.C.S., L.R.C.P., appointed Medical Officer for the Belton District of the Thorne Union. C. Killick Millard, M.B., B.Sc.Edin., appointed Medical Superintendent of the City Hospital, Birmingham. A. Campbell Munro, M.D., D.Sc., appointed Medical Officer of Health for the Burgh of Port Glasgow. H. W. Pooler, L.R.C.P., M.R.C.S., L.S.A., appointed Medical Officer and Public Vaccinator to the No. 4 District of Aston (Bir-

### THE STUDENT OF MEDICINE—*continued.*

mingham) Parish. Rhys B. Rees, L.S.A., appointed Medical Officer to the Malden Road and Haverstock Hill Provident Dispensary. C. Sanders, M.B.Lond., M.R.C.S.Eng., appointed Medical Officer, for the West Ham District, to the London and India Docks Joint Committee. W. St. Clair Symmers, M.B., appointed Assistant Bacteriologist in the British Institute of Preventive Medicine. J. T. Thomas, L.R.C.P.Lond., M.R.C.S. Eng., appointed Medical Officer of Health for the Camborne Urban District. T. W. Thomas, M.R.C.S.Eng., L.S.A., appointed Medical Officer for the Rudry District of the Rudry Union. C. F. Wightman, F.R.C.S.Eng., appointed Senior House Surgeon to the Bolton Infirmary. W. T. D. Allen, M.B., B.Ch., B.A.O. Royal University, Ireland, appointed Assistant Medical Officer at the Parish Infirmary, Brownlow Hill, Liverpool. E. W. P. Baines, M.B., B.S.Durham, appointed for 1896 to the post of Registrar at the Hospital for Women, Soho Square. J. B. Bawden, M.D.Edin., appointed Medical Officer for the Roche and St. Dennis (No. 2) District of the St. Austell Union. A. H. Cheatle, F.R.C.S., appointed Surgeon to the Royal Ear Hospital, Soho. W. S. Colman, M.D. Lond., M.R.C.P., appointed Assistant Physician to the National Hospital for the Paralysed and Epileptic, Queen Square, Bloomsbury. Mr. Couch, appointed Medical Officer for the Breage District of the Helston Union. R. Farrar, M.D.Oxon., M.R.C.S., L.R.C.P.Lond., appointed Surgeon to the Stamford, Rutland, and General Infirmary. C. Fenwick, L.R.C.P., L.R.C.S.Edin., appointed Medical Officer for the Cheriton Fitzpaine District of the Crediton Union. A. E. Griffin, M.A.Cantab., M.R.C.S.Eng., L.R.C.P.Lond., appointed House Surgeon to the Middlesex Hospital. A. W. Hall, L.R.C.P., F.R.C.S.Edin., Surgeon to ss. "Umlazi," Coolie Immigration Department, Natal Government. C. E. Hodgson, L.R.C.P., L.R.C.S.I., appointed Resident Clinical Assistant to the City Asylum, Birmingham. W. Newman,

### VACANCIES.

The following vacancies are announced this week, the amount of salary in each case being given after the name of the institution:—

#### Resident Medical Officers.

Ancoats Hospital, Manchester.—Resident Junior House Surgeon. Salary £50, with board and washing. Applications to Alex. Forrest, Honorary Secretary, by March 31st.

Bradford Infirmary.—Dispensary Surgeon and also Junior House Surgeon. Salary for the former £100 per annum, with board and residence; and for the latter £50, with board and residence. Candidates must be unmarried and doubly qualified. Applications endorsed "Dispensary Surgeon" and "Junior House Surgeon," to Wm. Maw, Secretary, by March 30th.

Counties Asylum, Garlands, Carlisle.—Junior Medical Assistant. Salary £80 a year, with board. Applications to Dr. Campbell, Garlands, Carlisle, by March 28th.

General Hospital, Birmingham.—Two Assistant House Surgeons; must possess surgical qualifications. No salary, but residence, board, and washing provided. Applications to Howard J. Collins, House Governor, by March 28th.

London Homœopathic Hospital, Great Ormond Street Bloomsbury, W.C. —Resident Medical Officer; legally qualified, and be, or become, a member of the British Homœopathic Society. Appointment for six months. Salary at the rate of £100 per annum, with board and apartments. Applications, with copies of 20 testimonials, to the Secretary-Superintendent by March 31st.

London Homœopathic Hospital, Great Ormond Street, Bloomsbury, W.C. —Assistant Resident Medical Officer; legally qualified, and be, or become, a member of the British Homœopathic Society. Appointment for six months. Salary at the rate of £40 per annum, with board and apartments. Applications, with copies of 20 testimonials, to the Secretary-Superintendent by March 31st.

London Lock Hospital, Harrow Road, W.—House Surgeon at the Male Hospital for twelve months. Salary £50, with board, lodging, and washing. Assistant House Surgeon at the Female Hospital. Board, lodging, and washing. Applications to the Secretary by March 31st.

Rotherham Hospital and Dispensary.—Assistant House Surgeon. Appointment for six months. No salary, but board, lodging, and washing provided. Applications to the House Surgeon by April 3rd.

Sunderland Infirmary.—House Surgeon; doubly qualified. Salary £80, rising £10 annually to £100, with board and residence. Applications to the Chairman of the Medical Board by April 2nd.

Worcester General Infirmary.—Assistant House Surgeon and Dispenser; unmarried. Salary £70 per annum, with board, residence, and washing. Appointment tenable for not more than two years. Applications to the Secretary by March 30th.

York Dispensary.—Resident Medical Officer; unmarried. Salary £150 a year, with furnished apartments, coal, and gas. Applications to Mr. W. Draper, De Grey House, York, by March 31st.

#### Non-Resident Medical Officers.

Borough of Sunderland.—Medical Officer of Health for the Borough and Port and Public Analyst. Salary in all £525 per annum. Will be debarred from private practice, and be required to devote his whole time to the duties. Must be qualified to practise medicine, surgery, and midwifery. Applications, endorsed "Applications for appointment of Medical Officer of Health and Public Analyst," to F. M. Bowey, Town Clerk, Town Hall, Sunderland, by March 31st.

Brighton Throat and Ear Hospital, 23, Queen's Road, Brighton.—Non-resident House Surgeon. Salary at the rate of £52 per annum. Applications to the Secretary by March 31st.

Charing Cross Hospital.—Assistant Surgeon; must be F.R.C.S.Eng. Applications to Arthur E. Reade, Secretary, by March 25th.

Royal College of Physicians.—Milroy Lecturer. Applications to the Registrar, Royal College of Physicians, Pall Mall East, by April 9th.

University College, Bristol, Faculty of Medicine.—Medical Tutor. Salary £125. Applications to the Dean by March 31st.

Great Ormond Street, Bloomsbury, W.C.

Secretary-Superintendent.

### THE 2/6 SERIES OF NURSING MANUALS.

**HOW TO BECOME A NURSE: AND HOW TO SUCCEED.** A complete guide to the nursing profession for those who wish to become Nurses, and a useful book of Reference for Nurses who have completed their training and seek employment. Compiled by HONNOR MORTEN, Author of "The Nurses' Dictionary," &c. Third and Revised Edition. Demy 8vo, 209 pp., profusely Illustrated with Copyright Portraits and Drawings.

"To those who are frequently appealed to by girls, or young women, as to the steps they should take to become nurses, this book of Miss Morten's must prove a perfect godsend."—*British Medical Journal.*

**THE NURSES' DICTIONARY OF MEDICAL TERMS AND NURSING TREATMENT.** Compiled for the use of Nurses, by HONNOR MORTEN. Containing descriptions of the principal Medical and Nursing Terms and Abbreviations, Instruments, Drugs, Diseases, Accidents, Treatments, Physiological Names, Operations, Foods, Appliances, &c., &c., encountered in the Ward or Sick-room. Third and Revised Edition (25th thousand). Demy 16mo, suitable for the apron pocket, handsomely bound in handsome leather, gilt, price 2s. 6d. net, or in cloth, price 2s.

"Should be at the disposal of every nurse."—*Birmingham Medical Review.*

**HOSPITAL SISTERS AND THEIR DUTIES.** By EVA C. LUCKES, Matron to the London Hospital. The favourable reception accorded to the first two editions of this useful and interesting work, and the regular demand for copies of it, encourage the publishers to place this third edition, greatly improved and enlarged, before the Institutions. The experience and position of its author entitle it to authority, and every Matron, Sister, or Nurse who aspires to become a Sister, should read the advice and information contained in its pages. Third Edition. Enlarged and partly rewritten, crown 8vo, cloth gilt, about 200 pp.

**MENTAL NURSING.** A Text-book specially designed for the instruction of Attendants on the Insane. By WILLIAM HARDING, M.D., M.R.C.P. The want of a complete book for the instruction of Asylum Attendants has been long felt, and it is with confidence that the publishers recommend this work to the managers of all Institutions for the Insane. Second Edition, Enlarged, demy 8vo, profusely illustrated, nearly 200 pp., cloth.

**SPINAL CURVATURE AND AWKWARD DEPORTMENT:** Their Causes and Prevention in Children. By Dr. GEORGE MULLER, Professor of Medicine and Orthopædics, Berlin. English Edition, edited and adapted by RICHARD GREENE, F.R.C.P. Crown 8vo, cloth gilt, with many Plates and Illustrations.

"Dr. Müller's little book is an able essay upon the effect produced by exercise, attitude, and movement upon the growth and development of the body. He further gives directions which any intelligent parent or teacher could carry out with the help of very simple apparatus, showing how to avoid or, in early cases, to cure certain malformations which, in the majority of cases, arise either from the neglect of very simple hygienic rules or from carelessness."—*Daily Chronicle.*

**THE MOTHER'S HELP AND GUIDE TO THE DOMESTIC MANAGEMENT OF CHILDREN.** By P. MURRAY BRAIDWOOD, M.D., formerly Senior Medical Officer to the Wirral Hospital for Sick Children. Crown 8vo, cloth gilt.

"The book is altogether admirable."—*Manchester Courier.*

London: THE SCIENTIFIC PRESS (Limited) 428, STRAND, W.C.

### "THE HOSPITAL"

AN INDEPENDENT JOURNAL OF
**The Medical Sciences**
AND
**Hospital Administration,**
WITH WHICH IS ISSUED WEEKLY
A SPECIAL
### SUPPLEMENT FOR NURSES.

PUBLISHED EVERY SATURDAY.
PRICE TWOPENCE.

Matrons, Sisters, or Nurses will find in THE HOSPITAL a chronicle of the latest Nursing News, Practical Articles of the highest value to those who wish to keep themselves abreast with the times, and brightly written articles from Nurses all over the world.

SUBSCRIPTIONS CAN COMMENCE AT ANY TIME.

### TERMS OF SUBSCRIPTION.

### WEEKLY ISSUE.

| | Great Britain. | Foreign and Colonial. |
|---|---|---|
| For One Year ... ... | 10s. 6d. ... | 15s. 2d. |
| For Six Months ... ... | 5s. 3d. ... | 7s. 7d. |
| For Three Months... ... | 2s. 8d. ... | 3s. 10d. |

### MONTHLY PARTS.

| | Post Free to any Part of the World. |
|---|---|
| For One Year ... ... | 8s. 6d. |
| For Six Months ... ... | 4s. 3d. |
| For Three Months ... ... | 2s. 2d. |

Postal Orders and Cheques should be made payable to "The Scientific Press, Limited."

N.B.—In order to prevent delay, all business communications should be addressed to "THE MANAGER" only, and not to any person individually.

### CHANGE OF ADDRESS.

In event of change of address, notice should be forwarded to the Publishers by the Saturday previous to date of publication, and the old address should also be given.

"THE HOSPITAL" can be obtained of all Booksellers and Newsagents in the United Kingdom, and at all the Railway Stalls, of Messrs. W. H. Smith and Son; and also from the following Agents in the Colonies and Abroad:—

PARIS: Librairie Galignani, 224, Rue de Rivoli.
BERLIN: Messrs. A. Asher and Co.
LEIPSIG: Mr. F. A. Brockhaus.
NEW YORK: The International News Co.
MONTREAL: The Montreal News Co.
TORONTO: The Toronto News Co.
MELBOURNE, SYDNEY, ADELAIDE, and ZEALAND: Messrs. Gordon and Gotch.
INDIA: At all the Railway Bookstalls and Agencies of Messrs. A. H. Wheeler and Co.
SOUTH AFRICA: Messrs. Juta, Cape Town.

PUBLISHING OFFICES: 428, STRAND, LONDON, W.C.

*In the Press. Price 5s.*

### BURDETT'S OFFICIAL NURSING DIRECTORY

A DIRECTORY, NOT A REGISTER.

THE HOSPITAL states: "It is well to bear in mind the difference and distinction which exists between a register and a directory. A register implies rights, and suggests that those who are not upon it are in some way or another inferior beings. But a directory is another matter. A directory gives as rights; insertion in it implies no adherence to any particular party or any particular opinion, and in no way interferes with entry on the register of any association, or the list of any institute, to which the individual may belong. All that a directory professes to do is to give information. The 'Medical Register' is not a mere list of qualified medical men; it is admission to the Register, which itself makes him qualified. For years, perhaps even now, 'Churchill's Medical Directory' contained the names of medical men who, so far as public appointments were concerned, were unqualified; it also contains the names of homœopathists, whom four out of every five medical men will not meet; and it certainly contains the names of many for whose moral character we should not like to vouch. But it gives full and accurate information about them all, and for the sake of that information it is constantly consulted both by the public and the doctors."

**The above work will be ready shortly, and those Nurses who have not already sent in their names should at once apply for a form, which can be obtained upon application to the Publishers,**

THE SCIENTIFIC PRESS (Lim.), 428, Strand, London, W.C.

Just Published. 8th Edition. Greatly enlarged and thoroughly revised. Crown 8vo., cloth gilt, 3s. 6d.

**NURSING: Its Theory and Practice,** being a Complete Text-book of Medical, Surgical, and Monthly Nursing. By PERCY G. LEWIS, M.D., M.R.C.S., L.S.A., &c., &c.

To this edition have been added three chapters on Monthly Nursing, together with additions to the chapters on Blood Diseases, on Antiseptics, on the Nursing of Children, and on Private Nursing. Under these headings will be found a short account of the new Aseptic Treatment, the Antitoxin and Serum Treatments, and the Treatment by Thyroid Extracts.

These additions, combined with the revision throughout of the text, render this work the best and most complete Text-book on Nursing in the market, and strengthen its position as a Classic in the Training Schools of the World.

"We have read the book with interest, and can warmly recommend it as a nursing manual. . . . Full of useful hints and information."—*Birmingham Medical Review.*

Just Out. With numerous Illustrations, demy 8vo., blue buckram, gilt, 3s. 6d.

**DIET IN SICKNESS AND IN HEALTH.**

By Mrs. ERNEST HART, formerly Student of the Faculty of Medicine of Paris, and of the London School of Medicine for Women. With an Introduction by Sir HENRY THOMPSON, F.R.C.S., M.B.Lond.

SIR HENRY THOMPSON, in his Introduction, says: "I do not hesitate to express my opinion that the present volume forms a handbook to the subject, which will not only interest the dietetic student, but offer him, within its modest compass, a more complete epitome thereof than any work which has yet come under my notice."

"Mrs. Hart speaks not only with the authority derived from experience, but with the ease and freedom of an expert. We have perused this book with great pleasure, and feel sure that many will find in it much to lengthen the days of those whose digestion is enfeebled."—*The Hospital.*

Second Edition (with a New Chapter on the History of Infant Feeding by Artificial Means in the Early Ages, Illustrated with Coloured and other Plates). Crown 8vo., cloth gilt, with 17 Plates and many Illustrations in the Text. Price 5s.

FACSIMILE AUTOGRAPH LETTERS FROM THE LATE SIR ANDREW CLARK, M. PASTEUR, &c., &c.

**INFANT FEEDING BY ARTIFICIAL MEANS:** A Scientific and Practical Treatise on the Dietetics of Infancy. By S. H. SADLER, Author of "Suggestions to Mothers," "Management of Children," "Education."

"Mrs. Sadler's book deals with the question of the artificial feeding of infants, and contains a very useful collection of the views of the best-known English authorities upon the subject. The truly terrible ignorance displayed by mothers, especially amongst the poor, upon the subject of infant feeding is answerable for an infant mortality as great as to be appalling."—*Daily Chronicle.*

London: THE SCIENTIFIC PRESS (LIMITED), 428, Strand, W.C.

**NURSING BOOKS AT FULL DISCOUNT.**

*LIST POST FREE.*

The Theory and Practice of Nursing (Percy Lewis, M.D.), 3/6 for 2/8.

The Nurse's Dictionary (Honnor Morten), 2/- for 1/6.

The Nurse's Case Book, 6d. for 3½d.

